# Supplementary material for: Dynamic transcriptomic profiles of zebrafish gills in response to zinc supplementation
Source: BMC Genomics. 2010 Oct 11;11:553. doi: 10.1186/1471-2164-11-553 (PMC3091702; doi:10.1186/1471-2164-11-553)
Supplement: Additional file 2 — Interactive Direct Interaction Network representing the molecular interactions between zinc, copper, iron, calcium and proteins encoded by transcripts changed by zinc supplementation. Mini web-site containing index.html and hyperlinked pages in subdirectory describing a Direct Interaction Network automatically generated based on curated interactions contained within the proprietary PathwayArchitect database. Ovals represent proteins and the circles symbolize metal ions. Objects are coloured by their abundance in zebrafish at the time-point they were significantly different from the control is a scale from -4 fold (dark green) to +4 fold (dark red). Where significant differences were found at more than one time-point, the colour overlay shows expression at the first instance. Dark blue squares denote 'binding', and light blue squares 'expression'; green squares stand for 'regulation', green diamonds for 'metabolism', and green circles for 'promoter binding'. Arrow heads indicate directionality of the interaction where annotated. All nodes and edges can be further interrogated by selecting the relative area of the image. [file 1471-2164-11-553-S2.zip › PathwayArchitect Zn xs DIN/143516.html]

# PROTEIN: ZNF384

|  |  |
| --- | --- |
| Name | ZNF384 |
| Type | PROTEIN |
| Description | zinc finger protein 384 |
| Note | This gene contains long CAG trinucleotide repeats coding consecutive glutamine residues. The gene product may function as a transcription factor, with a potential role in the regulation of neurodevelopment or neuroplasticity. Studies in mouse suggest that nuclear matrix transcription factors (NP/NMP4) may be part of a general mechanical pathway that couples cell construction and function during extracellular matrix remodeling. Multiple transcript variants have been detected for this gene, but their full-length natures have not been determined. |
| Alias | Nmp4 |
|  | nuclear matrix transcription factor 4 |
|  | ZNF384 |
|  | Nuclear matrix transcription factor 4 |
|  | Znf384 |
|  | nuclear matrix protein 4 |
|  | Ciz |
|  | Cas-interacting zinc finger protein |
|  | CAGH1A |
|  | Cas- associated zinc finger protein |
|  | CIZ |
|  | TNRC1 |
|  | CAGH1 |
|  | NP |
|  | trinucleotide repeat containing 1 |
|  | NMP4 |
|  | CAG repeat protein 1 |
|  | Cas-associated zinc finger protein |


---

|  |  |
| --- | --- |
| GO Component | nucleus |
|  | focal adhesion |


---

|  |  |
| --- | --- |
| GO ID | GO:0005634 |
|  | GO:0003677 |
|  | GO:0005925 |
|  | GO:0006355 |
|  | GO:0046872 |
|  | GO:0042156 |
|  | GO:0006913 |
|  | GO:0006350 |
|  | GO:0008270 |
|  | GO:0017124 |


---

|  |  |
| --- | --- |
| Connectivity | 43 |


---

|  |  |
| --- | --- |
| Entrez ID | 171017 |
|  | 171018 |


---

|  |  |
| --- | --- |
| Agilent ID | A\_44\_P374902 |
|  | A\_44\_P491653 |
|  | A\_14\_P104125 |
|  | A\_43\_P13192 |
|  | A\_23\_P204355 |
|  | A\_14\_P130304 |
|  | A\_14\_P122100 |


---

|  |  |
| --- | --- |
| Cellular Localization | Nucleus |
|  | Organelle |
|  | Cell |
|  | Plasma membrane |
|  | Membrane |


---

|  |  |
| --- | --- |
| Pathway | Zn xs inventory |
|  | Zn xs DIN |


---

|  |  |
| --- | --- |
| GO Process | nucleocytoplasmic transport |
|  | regulation of transcription, DNA-dependent |
|  | transcription |


---

|  |  |
| --- | --- |
| UniGene | Rn.9637 |
|  | Hs.103315 |


---

|  |  |
| --- | --- |
| Affymetrix Probeset ID | 1370175\_a\_at |
|  | 212369\_at |
|  | 38648\_at |
|  | 45473\_at |
|  | 73415\_at |
|  | Hs.103315.1.S1\_3p\_at |
|  | rc\_AI177799\_at |
|  | AA091467\_s\_at |
|  | RC\_AA132032\_s\_at |
|  | RC\_T95280\_r\_at |


---

|  |  |
| --- | --- |
| GO Function | SH3 domain binding |
|  | zinc-mediated transcriptional activator activity |
|  | DNA binding |
|  | zinc ion binding |
|  | metal ion binding |


---

|  |  |
| --- | --- |
| Nucleotide | AF216805 |
|  | AB070238 |
|  | U80738 |
|  | AF216804 |
|  | BC053361 |
|  | AK095734 |
|  | AF216806 |
|  | NM\_133476 |
|  | NM\_133429 |
|  | AF216807 |
|  | AB019281 |


---

|  |  |
| --- | --- |
| Protein | AAG40582 |
|  | NP\_596920 |
|  | BAB85125 |
|  | AAG40584 |
|  | AAH53361 |
|  | Q9EQJ4 |
|  | BAC04618 |
|  | AAG40583 |
|  | NP\_597733 |
|  | Q8TF68 |
|  | AAG40585 |
|  | BAA89664 |
|  | AAB91437 |


---

|  |  |
| --- | --- |
| Organism | Mammal |


---

|  |  |
| --- | --- |
| Location | chromosome 12 (Homo sapiens) |
|  | chromosome 4, 4q42 (Rattus norvegicus) |


---

|  |  |
| --- | --- |
